# Lateral Electronic Junction of a Single Ultrathin Silicon Induced by Interfacial Dipole of Self‐Assembled Monolayer

**DOI:** 10.1002/advs.202403970

**Published:** 2024-09-09

**Authors:** Junghyup Han, Won Hyung Lee, Junwoo Park, Huding Jin, Yong Hyun Cho, Seungyeon Yu, Lianghui Li, Jaewon Lee, Gunhoo Woo, Taesung Kim, Youn Sang Kim

**Affiliations:** ^1^ Department of Chemical and Biological Engineering College of Engineering Seoul National University 1 Gwanak‐ro, Gwanak‐gu Seoul 08826 Republic of Korea; ^2^ Program in Nano Science and Technology Graduate School of Convergence Science and Technology Seoul National University 1 Gwanak‐ro, Gwanak‐gu Seoul 08826 Republic of Korea; ^3^ Samsung SDI Co. Ltd. 130 Samsung‐ro, Yeongtong‐gu Suwon‐si Gyeonggi‐Do 16678 Republic of Korea; ^4^ Department of Chemistry Sogang University 35 Baekbeom‐ro, Mapo‐gu Seoul 04107 Republic of Korea; ^5^ Institute of Chemical Processes Seoul National University 1 Gwanak‐ro, Gwanak‐gu Seoul 08826 Republic of Korea; ^6^ Department of Chemical Engineering University of California Santa Barbara CA 93106 USA; ^7^ SKKU Advanced Institute of Nanotechnology Sungkyunkwan University (SKKU) 2066 Seobu‐ro, Jangan‐gu Suwon‐si Gyeonggi‐Do 16419 Republic of Korea; ^8^ Department of Nano Science and Technology Sungkyunkwan University 2066 Seobu‐ro, Jangan‐gu Suwon‐si Gyeonggi‐Do 16419 Republic of Korea; ^9^ School of Mechanical Engineering Sungkyunkwan University 2066 Seobu‐ro, Jangan‐gu Suwon‐si Gyeonggi‐Do 16419 Republic of Korea; ^10^ Department of Semiconductor Convergence Engineering Sungkyunkwan University 2066 Seobu‐ro, Jangan‐gu Suwon‐si Gyeonggi‐Do 16419 Republic of Korea; ^11^ Advanced Institute of Convergence Technology 145 Gwanggyo‐ro, Yeongtong‐gu Suwon‐si Gyeonggi‐Do 16229 Republic of Korea

**Keywords:** interface engineering, interfacial dipole, lateral electronic junction, self‐assembled monolayer

## Abstract

Interface engineering is pivotal for enhancing the performance and stability of devices with layered structures, including solar cells, electronic devices, and electrochemical systems. Incorporating the interfacial dipole between the bulk layers effectively modulates the energy level difference at the interface and does not significantly influence adjacent layers overall. However, interfaces can drastically affect adjoining layers in ultrathin devices, which are essential for next‐generation electronics with high integrity, excellent performance, and low power consumption. In particular, the interfacial effect is pronounced in ultrathin semiconductors, which have a weak electric field screening effect. Herein, the substantial interfacial impact on the ultrathin silicon is shown, the p‐ to n‐type inversion of the semiconductor solely through the deposition of a self‐assembled monolayer (SAM) without external bias. The effects of SAMs with different interfacial dipoles are investigated by using Hall measurement and surface analytic techniques, such as UPS, XPS, and KPFM. Furthermore, the lateral electronic junction of the ultrathin silicon is engineered by the regioselective deposition of SAMs with opposite dipoles, and the device exhibits rectification behavior. When the interfacial dipole of SAM is manipulated, the rectification ratio changes sensitively, and thus the fabricated diode shows potential to be developed as a sensing platform.

## Graphical abstract

Lateral electronic junction of a single ultrathin silicon is engineered through the regioselective deposition of self‐assembled monolayers (SAMs) with opposite dipoles, and the surface‐modified silicon exhibits the rectification behaviors with the ideality factor of 1.64. The diode is developed through interface modification, which modulates the electronic properties of the ultrathin silicon with the interfacial dipole layer.

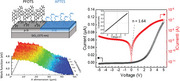

## 1 Introduction

Interfacial phenomena play an essential role in operating devices with layered structures, such as solar cells,^[^ ^1^, ^2^, ^3^ ^]^ molecular electronic devices,^[^ ^4^, ^5^, ^6^ ^]^ and energy storage/harvesting devices,^[^ ^7^, ^8^, ^9^, ^10^ ^]^ which possess electrochemical and electronic interfaces. Therefore, optimal interface engineering techniques for layered devices have been developed based on a comprehensive understanding of interfacial phenomena. Remarkable device performance and stability were achieved by addressing the challenges at the interface, such as energy band misalignment and unstable solid‐electrolyte interphase.^[^ ^11^, ^12^, ^13^ ^]^ Incorporating an interfacial dipole, one of the most representative interface engineering techniques, facilitates precise control over the electronic structure at the interface. Consequently, this modulation effectively enhances charge carrier transport within solar cells while impeding such transport within diodes.^[^ ^14^, ^15^, ^16^, ^17^ ^]^ Unlike the significant effect at the interface, the influence of the interfacial dipole on the adjacent layer overall is minimal in devices composed of bulk layers with micrometer thickness, and it is generally considered negligible. On the other hand, this interfacial influence is no longer trivial for devices comprised of ultrathin layers with nano‐ and atomic‐scale thickness, which are essential for developing next‐generation electronics with high integrity, excellent performance, and low power consumption. The characteristics of 2D materials with atomically thin structures were steered by modifying the surface of the substrate on which the 2D materials are transferred rather than by reforming the materials themselves.^[^ ^18^, ^19^, ^20^, ^21^, ^22^ ^]^ In particular, ultrathin layers with semiconducting properties are enormously affected by the interfacial dipole due to their weak electric field screening effect. Thus, the physical and electronic properties of ultrathin semiconductors not only originate from their intrinsic atomic structure but are also drastically influenced by the surrounding environments, involving the interface and the adjoining layers.^[^ ^23^, ^24^, ^25^, ^26^ ^]^ Even in semiconductors with tens of nanometers thickness, which are mainly used in current silicon‐based industries, their electronic properties can be significantly impacted by the surrounding interfaces. However, this prominent interfacial effect is still underestimated.

In this study, we show the substantial impact of the incorporated interfacial dipole layers on the ultrathin semiconductor, which is the p‐ to n‐type inversion of the silicon with 50 nm thickness solely by the SAM deposition without any external bias. Through the Hall measurement and surface analytic techniques such as UPS, XPS, and KPFM, we verified the drastic effects of SAM deposition on the electronic properties of the ultrathin semiconductors according to the packing density of SAM and the SAM species with opposite dipole direction. Unlike the conventional silicon with micrometer thickness, the control on the type and carrier concentration of the ultrathin silicon was rendered by modulating the direction and strength of the interfacial dipole of SAM without direct injection of chemical impurity into the atomic structure of the semiconductor. Furthermore, we show that the lateral electronic junction between the SAM‐induced n‐ and p‐type region in a single ultrathin silicon can be engineered by regioselective patterning of SAMs possessing dipole moments in opposite directions. The ultrathin silicon, which initially had the ohmic behavior, exhibited rectifying behavior reminiscent of a p‐n diode after the regioselective SAM treatment. Since the conventional impurity doping techniques in CMOS technology have difficulty in the inconsistent device performance due to the random distribution of dopants in nanoscale semiconductors, these results suggest that an interface engineering approach could serve as a simple alternative to control the type and carrier density of the ultrathin semiconductors. The diode‐like device induced by the interfacial dipole of SAMs also has the potential as a pH‐detecting sensor since the rectification ratio sensitively changes according to the electrolyte pH due to the pH‐dependent amine groups of SAM. Therefore, the ultrathin silicon treated with the regioselective SAM deposition could be developed as a new sensing platform by introducing various probe‐grafted SAMs, of which interfacial dipole significantly responds to the specific interaction with target molecules.

## 2 Results and Discussion

### 2.1 Impacts of Incorporating Interfacial Dipole on the Ultrathin Semiconductor

**Figure 1:**
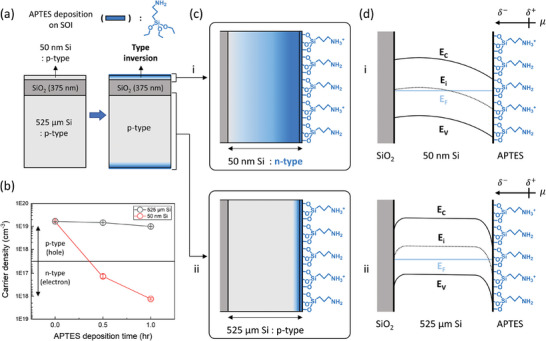
Impacts of incorporating interfacial dipole layer on the electronic properties of the 50 nm silicon. a) A schematic image of the side view of the silicon‐on‐insulator (SOI) before and after (3‐Aminopropyl)triethoxysilane (APTES) deposition. b) Carrier density change of silicon with 50 nm and 525 µm thickness according to the APTES deposition time, measured through Hall measurement. In the case of the 50 nm silicon, the p‐ to n‐type inversion occurs, and therefore, the majority carrier of silicon is changed from hole to electron. (c) Influence of the interfacial effect on the silicon with i) 50 nm and ii) 525 µm thickness, which is depicted as a gradation. d) Energy band diagram of the silicon with i) 50 nm and ii) 525 µm thickness, which are affected by the incorporation of the interfacial dipole layer, APTES.

To verify the substantial impacts of incorporating interfacial dipoles on the ultrathin semiconductor, we confirmed the change in the electronic property of ultrathin silicon induced by SAM deposition through the Hall measurement (**Figure** **1**). We used the silicon‐on‐insulator (SOI), which is composed of silicon with 50 nm and 525 µm thickness, and the buried SiO_2_ with 375 nm thickness, and (3‐aminopropyl)triethoxysilane (APTES) was deposited at each silicon surface. Silicon with a single crystalline structure was chosen as a semiconducting material since it is suitable for interpreting the interfacial effect based on classical semiconductor device theories. As an incorporated interfacial dipole layer, APTES is compatible with silicon and has a prominent electric dipole effect on the semiconductor emanating from the positive charges of surficial protonated amine groups of APTES. Moreover, investigating interfacial effects according to the direction and strength of the interfacial dipole is feasible by the facile and versatile physicochemical modification on amine groups of APTES, such as protonation/deprotonation, ion pairing, EDC‐NHS reactions, SN_2_ reactions, etc. Except for the different thicknesses of silicon, all other experimental conditions were set to be the same, such as APTES deposition conditions, specifications of silicon: p‐type (B‐doped), resistivity (1–20 ohm·cm), and orientation (<100>). Figure 1a shows the schematic image of the side view of the SOI before and after the APTES deposition on both silicon. We conducted Hall measurements to determine changes in the electronic properties of the silicon, such as the type and the majority carrier density of the semiconductor, which were induced by the APTES deposition, as delineated in Figure 1b. As APTES deposited, the hole density of 50 nm p‐type silicon drastically decreased, and then the p‐ to n‐type inversion occurred. On the other hand, the hole density of 525 µm p‐type silicon slightly decreased than that of 50 nm silicon, and the type inversion was not observed. These results indicate that while the influence of the interface is minor in bulk semiconductors, it is substantial in ultrathin semiconductors. Therefore, in contrast to the bulk semiconductor, when considering the characteristics of ultrathin semiconductors, it becomes imperative to consider the impacts of the interfaces along with the intrinsic properties of the material.

We depict the interfacial influence of APTES deposition as a gradation, which affects ultrathin silicon overall but has a minor effect on the bulk silicon in Figures 1c(i,ii), respectively. According to classical semiconductor theory, band bending occurs at the electronic band structure of semiconductors since they have the poor capability to screen the electric field induced by the surface charge owing to their low carrier density.^[^ ^25^ ^]^ This phenomenon at the bulk semiconductor has been considered locally confined at the interface. However, considering that the interfacial effect exerts enormous influence in the case of the ultrathin semiconductor, we illustrated that band bending occurs across the entire energy band structure of the ultrathin semiconductor in Figure 1d(i).^[^ ^23^ ^]^ Since the hole density of the p‐type silicon decreases when the APTES is deposited (Figure 1b), the Fermi level (*E*_F_) shifts toward the lowest energy level of the conduction band (*E*_C_). The region where *E*_F_ is higher than the intrinsic energy level (*E*_i_) widely distributes in the 50 nm p‐type silicon thereby leading to p‐ to n‐type inversion. Through the ARXPS depth profile, we confirmed that the Fermi level shift induced by the APTES deposition occurs up to a depth of 7.5 nm silicon, and the ARXPS results can partially support our illustration in Figure 1d(i) (Figure S1, Supporting Note 1, Supporting information). On the other hand, the semiconductor type of 525 µm silicon is maintained as a p‐type since the Fermi level shift occurs locally at the vicinity of the interface, as depicted in Figure 1d(ii). Therefore, in this study, we interpret the changes in electronic properties of 50 nm silicon caused by the interfacial dipole layer in terms of the fermi level shift rather than band bending.

### 2.2 Interfacial Dipole Direction‐Dependent Effect on the 50 nm Silicon

**Figure 2:**
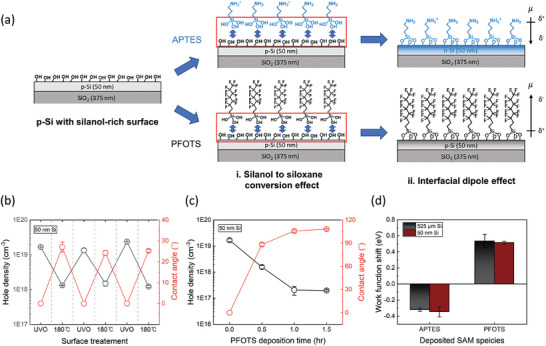
Interfacial dipole direction‐dependent electronic property changes of the 50 nm silicon. a) A schematic image of the procedure of SAM deposition and determinative factors affecting the electronic property of the ultrathin semiconductor. b) Change in the hole density and contact angle of 50 nm silicon by the iterative alternation of UVO treatment (30 min) and annealing process (180 °C for 1 h), which induce the surficial silanol‐to‐siloxane interconversion. c) Change in the hole density and contact angle of 50 nm silicon according to the PFOTS deposition time. d) Work function shifts of the 50 nm and 525 µm silicon resulted from the APTES and PFOTS deposition, which were confirmed by UPS.

To elucidate the effects of incorporating interfacial dipole dependent on the dipole moment direction of SAM molecules, we conducted comparative analyses of influences on the ultrathin silicon exerted by the deposition of (3‐Aminopropyl)triethoxysilane (APTES) and triethoxy(1H,1H,2H,2H‐perfluoro‐1‐octyl)silane (PFOTS) possessing dipole moments in opposite directions (**Figure** **2**). These analyses were based on the molecular structures of APTES and PFOTS, which have the same head group but different functional groups. Determinative factors affecting the electronic property of the semiconductor by the SAM deposition are illustrated in Figure 2a. One of the primary factors is the surficial silanol to siloxane conversion on the ultrathin silicon by the grafting process of the SAM. The other factor is the interfacial dipole effect originating from the deposited SAM molecules.

Before the SAM deposition, the UVO treatment for enhancing the packing density of SAM is conducted on the silicon surface, and thereby the siloxane bonds are broken and converted to silanol groups. The silanol‐rich surface exhibits a negatively charged surface, and the electric field effect caused by the surface charge induces hole accumulation in the vicinity of the silicon surface.^[^ ^27^ ^]^ As mentioned above (Figure 1), though the influence of the interfacial phenomenon, such as surface charge‐induced carrier accumulation/depletion, is generally confined to the interface, it could be substantial to the characteristics of adjoining layers in the case of the ultrathin semiconductor. A significantly high hole density of the UVO‐treated 50 nm silicon was measured as 1.68 × 10^19^ cm^‐3^, as shown in Figure 2b. During the SAM deposition, the silanol groups on the silicon surface are converted to siloxane bonds by condensation with the silanol anchoring groups of the SAM molecules, which were hydrolyzed from the ethoxy head groups of PFOTS and APTES. Then, the existing silanol‐induced hole accumulating effect on the silicon is reduced in both APTES and PFOTS deposition since those SAM molecules have the same head groups. Consequently, it was confirmed that each hole density equally tends to decrease when APTES and PFOTS are deposited, as shown in Figures 1b,c, respectively. These results could be supported by observing the hole density change of the 50 nm silicon when alternative surface treatments induce surficial silanol to siloxane conversion which is akin to what occurs during the SAM deposition. Specifically, we conducted annealing at 180 °C for 1 h to condense silanol groups and convert them to siloxane bonds on the silanol‐rich surface of silicon, which had been priorly treated with UVO for 30 min. As shown in Figure 2b, and Figure S2 (Supporting Information), the contact angle of the UVO‐treated silicon surface measured as ca. 0°, attributed to the abundance of hydrophilic silanol groups. In contrast, the contact angle of the annealed silicon surface exceeds 20°, which indicates that a few of the silanol groups were converted to siloxane bonds on the silicon surface, rendering the surface less hydrophilic. Upon the iterative alternation of these treatments, a consistent trend in contact angle variation was observed, which could be attributed to the reversible interconversion between surficial silanol and siloxane.^[^ ^28^ ^]^ At the same time, we iteratively verified a reduction in hole density with each instance of silanol‐to‐siloxane conversion on the silicon surface induced by annealing. Consequently, these results can support that surficial silanol to siloxane conversion occurred during the deposition of PFOTS and APTES, thereby reducing the hole‐accumulating effect of silanol.

As shown in Figure 2c, we confirmed that once the silicon surface is saturated with grafted PFOTS molecules, no further decrease in hole density occurs by observing the changes in hole density of 50 nm silicon according to the PFOTS deposition time. The PFOTS‐deposited silicon surface becomes more hydrophobic as PFOTS grafted, and it was corroborated by that the contact angle increases and then saturates at ca. 110°, which indicates the silicon surface is fully packed with PFOTS molecules (Figure S3, Supporting Information). Unlike the APTES deposition, the reduction in hole density with PFOTS deposition is not significant enough to induce p‐ to n‐type inversion. This difference in the carrier density change of 50 nm silicon is attributable to the different interfacial dipole effects originating from functional groups within the SAMs.^[^ ^29^ ^]^ We measured the work function shift (△ Φ) of the silicon induced by the SAM deposition through Ultraviolet photoelectron spectroscopy (UPS) analysis, which is a highly surface‐sensitive and elaborate technique for characterizing the interfacial dipole effect on the semiconductor. The results of △ Φ by the SAM deposition were interpreted in terms of Fermi level (*E*_F_), which is defined as *E*_F_ = *E*_VAC_ − Φ, where *E*_VAC_ denotes the energy level in the vacuum.^[^ ^30^ ^]^ As illustrated in Figure 2d, the deposition of APTES and PFOTS induced a negative and positive work function shift, respectively. Therefore, the Fermi level shifts toward the lowest energy level of the conduction band (*E*_C_) in the case of APTES and away from it in the case of PFOTS. These results indicated that the opposite interfacial dipole effect of APTES and PFOTS results in the decrease and increase in the hole density of the silicon, respectively.

Overall, we illustrate the SAM deposition‐induced electronic property change of silicon by comprehensively considering the main determinants: Silanol‐to‐siloxane conversion and interfacial dipole effects. Primarily, silanol groups on the silicon surface are condensed with the anchoring groups that both APTES and PFOTS have, and thereby, the surficial silanol‐induced hole accumulating effect decreases, resulting in the hole density decrease in both SAM deposition. Next, the opposite interfacial dipole effects of the APTES‐ and PFOTS‐deposited layers induce hole density to reduce and increase, respectively. Consequently, when APTES is deposited on the p‐type 50 nm silicon, the hole density significantly reduces and can be lower than the density of electron, which is the minority carrier of silicon thereby leading to p‐ to n‐type inversion. On the other hand, the hole density reduction of the p‐type 50 nm silicon by PFOTS deposition is less pronounced, and the majority carrier of the silicon is still maintained as a hole.

Additionally, we observed nearly the same magnitude of negative work function shift when we applied the same APTES treatment on 525 µm silicon as that on 50 nm silicon (Figure 2d). These results indicate that the interfacial dipole effects of the APTES layer exerted on both 50 nm and 525 µm silicon are nearly the same since the work function measured through the UPS analysis is from the silicon near the surface due to the low penetration depth of the UV light. Despite the all of same experimental conditions, involving APTES deposition, the specifications of silicon such as type, resistivity, and orientation, the enormous difference in carrier density reduction of 50 nm and 525 µm silicon (Figure 1b) can ultimately be attributed to the thickness of the semiconductor, which is the only different experimental condition. Therefore, these results in Figure 2 show again that interface engineering can have a substantial impact on the electronic characteristics of adjoining layers, especially in the case of ultrathin semiconductors.

### 2.3 Interface Engineering to Construct the Lateral Electronic Junction of the 50 nm Silicon

**Figure 3:**
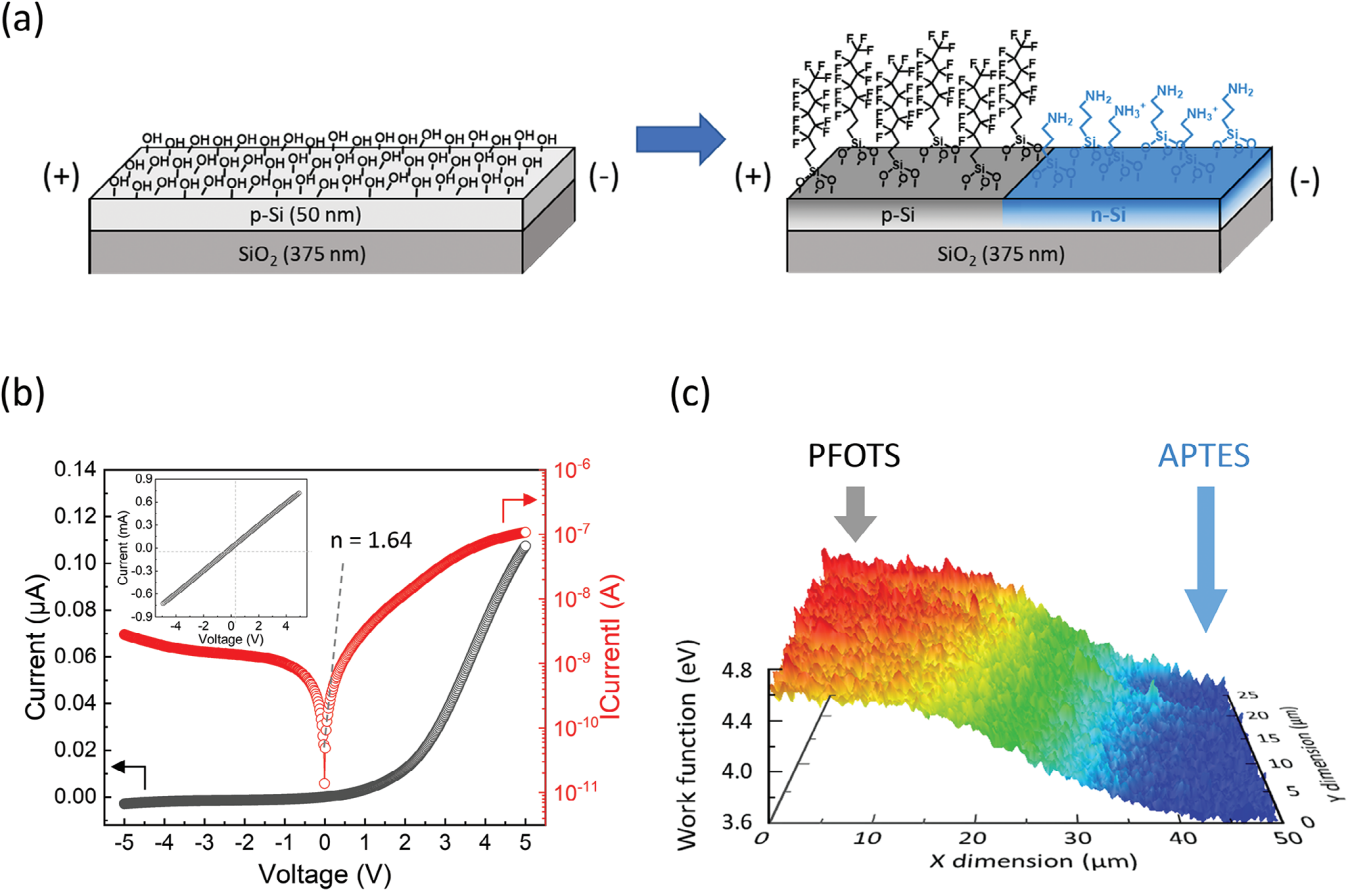
Interface engineering to construct the lateral electronic junction of the 50 nm silicon. a) A schematic image of the regioselective PFOTS and APTES deposition on the 50 nm silicon. b) IV characteristics of the PFOTS/APTES‐deposited 50 nm silicon exhibiting the rectifying behavior. The inset shows the IV characteristics of the 50 nm silicon before the regioselective deposition of PFOTS and APTES. The ideality factor (*n*) is calculated with an IV curve in a log scale. c) KPFM scanning image at the boundary between PFOTS‐ and APTES‐deposited surfaces of the PFOTS/APTES‐deposited silicon indicating the existence of the lateral electronic junction.

As illustrated in Figures 1 and 2, we showed the potential to modulate the electronic characteristics of ultrathin silicon via a simple interface engineering approach using SAM deposition. The semiconductor type of ultrathin silicon was determined by the introduced SAM species with opposite dipole direction, and the carrier density was modulated by controlling the packing density of the SAM. Furthermore, in **Figure** **3**, we present the approach to fabricating a diode, which is an essential unit of electronic devices, by engineering the lateral electronic junction of ultrathin silicon through the interface engineering technique rather than classical methods. As depicted in Figure 3a, we implemented a regioselective SAM deposition method to induce each p‐ and n‐type region separately at the single 50 nm silicon with an existing homogenous silanol surface (details are denoted in the Experimental Section).^[^ ^31^ ^]^ Consequently, the silicon surface was divided into the PFOTS‐deposited and APTES‐deposited regions, thereby forming a boundary between each SAM‐treated surface. As shown in Figure S4 (Supporting Information), the obvious difference in contact angles at the PFOTS‐ and APTES‐deposited surface was confirmed, corresponding to that on the surface packed with each SAM molecule. These results indicate the successful formation and preservation of each SAM‐deposited surface after the regioselective SAM deposition. Additionally, when a water droplet was introduced to the boundary of SAM‐treated surfaces, wetting occurred exclusively on the hydrophilic APTES‐deposited surface along the boundary, demonstrating the well‐formed boundary between PFOTS‐ and APTES‐deposited surfaces (Figure S4, Supporting Information).

To characterize the change in electrical properties of the 50 nm silicon by the regioselective SAM deposition, we conducted the current‐voltage (I‐V) measurements. As depicted in Figure 3b, the PFOTS/APTES‐deposited 50 nm silicon exhibited a rectifying behavior, which is reminiscent of a diode. We calculated the ideality factor from the following Shockley diode equation:

(1) $$I=I_{s}\left(e^{\frac{qV}{nkT}}-1\right)$$

where *I* is the diode current, *I*_S_ is the reverse bias saturation current, *q* is the electron charge, *V* is the voltage across the diode, *k* is the Boltzmann constant, *T* is the absolute temperature (K), and *n* is the ideality factor. The calculated ideality factor of 1.64 indicates that the PFOTS/APTES‐deposited 50 nm silicon operates well as a diode. Before the regioselective SAM deposition, the 50 nm silicon with a silanol‐rich surface exhibits linear ohmic behavior due to the homogenous surface, as shown in the inset of Figure 3b. These results indicate that the change in electrical characteristics of the silicon arose from the formation of a lateral electronic junction between p‐ and n‐type regions induced by the interfacial dipole of PFOTS and APTES layers, respectively. We conducted Kelvin probe force microscopy (KPFM) measurements to verify the presence of the lateral electronic junction of the PFOTS/APTES‐deposited 50 nm silicon.^[^ ^32^ ^]^

As depicted in Figure 3c, the mapping image describes the work function of the PFOTS/APTES‐deposited 50 nm silicon when scanning over the surface near the boundary of the PFOTS‐ and APTES‐deposited regions. The difference of work function in the PFOTS‐deposited region (*x* < 10 µm) and APTES‐deposited region (*x* > 40 µm) is ca. 0.9 eV, which is consistent with the results of UPS analysis (Figure 2d). Therefore, this result indicates that a lateral electronic junction between silicon regions with different Fermi levels exists under the boundary of the PFOTS‐ and APTES‐deposited surfaces (*x* = 10–40 µm). We fabricated the device operating like a diode solely through the surface treatments on the 50 nm silicon. Namely, modulating semiconductor properties and implementing electronic devices, which was previously achieved through internal dopant control in semiconductors has now been accomplished through the modification of the semiconductor interface. Therefore, an interface engineering approach has the potential to replace the traditional impurity doping technology used for constructing electronic devices if it ensures thermal stability, compatibility with existing CMOS processes, and preservation from the external environment using a protective layer.

### 2.4 Interfacial Dipole Strength‐Dependent Effect on the 50 nm Silicon and the Modulation of the Lateral Electronic Junction Through Ion Pairing

**Figure 4:**
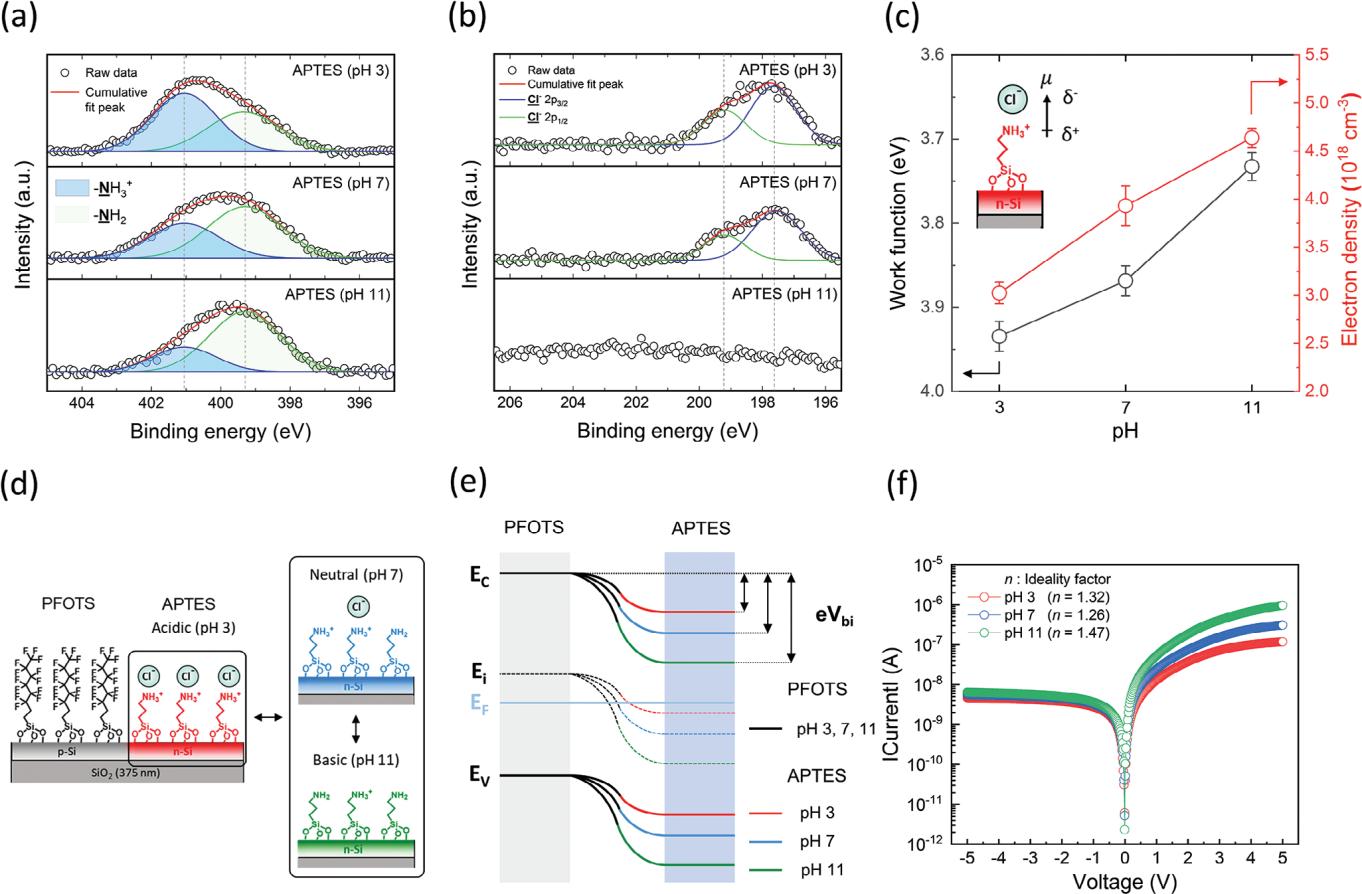
Interfacial dipole strength‐dependent electronic and electrical property change of the 50 nm silicon. a) N 1s XPS spectra, b) Cl 2p XPS spectra, c) electron density and work function of the n‐type APTES‐deposited 50 nm silicon treated with NaCl solutions of different pH levels (3, 7, 11). d) A schematic image of the PFOTS/APTES‐deposited 50 nm silicon with different surface states and corresponding electronic states when treated in different pH conditions (3, 7, 11). e) Energy band diagram of the formed lateral electronic junction of the PFOTS/APTES‐deposited 50 nm silicon. f) IV characteristics of the Cl^−^‐paired PFOTS/APTES‐deposited 50 nm silicon exhibiting the rectifying behaviors with different rectification ratios according to the pH of the treated NaCl solution. The ideality factor (*n*) is calculated with an IV curve in a log scale.

To elucidate the effect of the interfacial dipole according to its strength, we investigated the changes in the electronic properties of the n‐type APTES‐deposited 50 nm silicon through Hall measurement by modulating the ionic dipole of the SAM (**Figure** **4**). Ion pairing method was introduced to control the dipole moment of the APTES, and it was carried out by immersing the n‐type APTES‐deposited 50 nm silicon in 10 mm NaCl solutions with different pH (3, 7, 11) for 5 min followed by drying under a nitrogen stream.^[^ ^33^, ^34^ ^]^ Neutral primary amine (─NH_2_) and protonated amine (─NH_3_^+^) with positive charge coexist on the APTES‐deposited silicon surface, and the proportion of ─NH_3_^+^ groups increases as the solution in contact becomes more acidic. Chloride ions (Cl^‐^) in the NaCl solution form ion pairs with ─NH_3_^+^ groups of the APTES‐deposited silicon surface by Coulombic attraction thereby inducing ionic dipole at the APTES molecular layer. The strength of the newly formed ionic dipole is larger than the existing C–N^+^ bond dipole, and the direction of it points away from the surface, opposite to that of the C–N^+^ bond dipole. The interfacial dipole of the Cl^‐^‐paired APTES layer is composed of single electric dipole moments induced by ─NH_2_, ─NH_3_^+^, and Cl^‐^‐paired ─NH_3_^+^ groups, and its strength is dominantly affected by the ionic dipole, which possesses the largest dipole moment.^[^ ^33^ ^]^ Therefore, through the control on the amount of ─NH_3_^+^ groups that can pair with Cl^‐^ by treating with different pH solutions, we can modulate the interfacial dipole strength of the Cl^‐^‐paired APTES layer.

We conducted the X‐ray photoemission spectroscopy (XPS) analysis to verify the existence and relative amount of the ionic dipole at the Cl^‐^‐paired APTES layer. Figure 4a displays the N 1s spectra of the APTES‐deposited surface when treated with NaCl solutions of varying pH levels (3, 7, 11). The N 1s spectrum features peaks corresponding to the ‐NH_3_^+^ group (401.05 eV) and ‐NH_2_ group (399.3 eV) of the APTES‐deposited surface, indicating a successful APTES deposition. The area ratio of the peaks (─NH_3_^+^/─NH_2_) is 0.57, 0.39, and 0.27 at pH 3, 7, and 11, respectively, demonstrating a higher presence of ‐NH_3_^+^ groups when using more acidic solutions. Figure 4b presents the Cl 2p spectra of the same Cl^‐^‐paired APTES layer mentioned above. The doublet peaks of Cl 2p_3/2_ and Cl 2p_1/2_ emerged at pH 3 and 7 and were not found at pH 11, showing the absence of ion pairing of Cl^‐^ with ‐NH_3_^+^ group in a basic environment. The binding energy of Cl 2p_3/2_ (199.2 eV) and Cl 2p_1/2_ (197.6 eV) originates from the chloride ion (Cl^‐^), suggesting that the paired Cl^‐^ with ‐NH_3_^+^ group remains on the APTES‐deposited surface. Additionally, the quantification of Cl and N was performed to compare the relative amount of paired Cl^‐^ on the APTES layer treated with NaCl solutions of varying pH levels (3, 7, 11). The area ratio of the spectra (Cl 2p / N 1s) at pH 3 is 1.78 times greater than that at pH 7, indicating that more paired Cl^‐^ exist in a more acidic environment due to the more NH_3_^+^ groups available to pair with Cl^‐^. Therefore, these results imply that the effect of the interfacial dipole according to its strength is possible to compare through the ion pairing method.

As depicted in Figure 4c, the newly‐formed ionic dipole points away from the surface and induces an electron‐depleting effect on the n‐type APTES‐deposited 50 nm silicon, consequently shifting the Fermi level towards the highest energy level of the valence band (*E*_V_).^[^ ^35^ ^]^ When the n‐type APTES‐deposited 50 nm silicon was treated with NaCl solutions of varying pH levels (3, 7, 11), the electron density was measured as 3.02, 3.93, and 4.64 × 10^18^ cm^‐3^, respectively, through Hall measurements, and the work function was measured as 3.93, 3.87, and 3.73 eV, respectively, through KPFM. These results show the possibility of controlling the carrier density of an ultrathin semiconductor by adjusting the interfacial dipole strength. We also demonstrated that the lateral electronic junction of the PFOTS/APTES‐deposited 50 nm silicon can be modulated through the control of the interfacial dipole strength of the APTES‐deposited layer, thereby changing its electrical characteristics.

Figure 4d shows the schematic image of the PFOTS/APTES‐deposited 50 nm silicon with different surface states and corresponding electronic states when the silicon was treated with NaCl solutions of varying pH levels (3, 7, 11). This image is depicted based on the results of XPS analysis and Hall measurement on the n‐type APTES‐deposited 50 nm silicon (Figure 4a–c) and the p‐type PFOTS‐deposited 50 nm silicon with the same treatment with NaCl solutions. In the XPS analysis on the p‐type PFOTS‐deposited 50 nm silicon, there was no peak observed in the Na 1s and Cl 2p spectra at all pH conditions of the NaCl solution (Figure S5, Supporting Information). These results imply that no ion pairing occurred at the PFOTS‐deposited surface. We depicted the energy band diagram of the formed lateral electronic junction of the PFOTS/APTES‐deposited 50 nm silicon based on the results of KPFM and Hall measurement (Figure 4e). A depletion region is formed between the PFOTS‐deposited silicon where the energy band is invariant according to the pH condition and the APTES‐deposited silicon where the Fermi level shifts dependent on the pH condition. Therefore, the resulting built‐in potential (e*V*_bi_) of the PFOTS/APTES‐deposited 50 nm silicon is determined by the pH of treating NaCl solutions. When the PFOTS/APTES‐deposited 50 nm silicon is treated with a more acidic solution, a smaller e*V*_bi_ is formed since the difference in Fermi level position between the PFOTS‐deposited silicon region and APTES‐deposited silicon region is smaller compared to when treated with a basic solution. Figure 4f shows the I‐V characteristics of the PFOTS/APTES‐deposited 50 nm silicon treated with the NaCl solutions of varying pH levels (3, 7, 11), exhibiting diode behaviors with rectification ratios of 25, 55, and 149, respectively where the rectification ratio is defined as *I*_(*V* = 5 V)_ / − *I*_(*V* =  − 5 V)_. Since the rectification ratio is directly related to the built‐in potential, this difference of rectification could arise from the varying e*V*_bi_ in the lateral electronic junction of the PFOTS/APTES‐deposited 50 nm silicon, depending on the solution pH. Consequently, it was confirmed that the electrical property of the PFOTS/APTES‐deposited 50 nm silicon was determined by the ionic dipole at the APTES‐deposited surface, which induces the Fermi level shift of the APTES‐deposited silicon region thereby changing the e*V*_bi_. We conclusively demonstrated that controlling the interfacial dipole of the APTES layer through the ion pairing method can modulate the lateral electronic junction thereby changing the electrical characteristics of the PFOTS/APTES‐deposited 50 nm silicon.

Furthermore, the PFOTS/APTES‐deposited 50 nm silicon has the potential to be developed as a pH‐detecting sensor considering the rectification ratio changes sensitively according to the pH of the treating solution. When the ultrathin silicon treated with regioselective SAM deposition is composed of various probe‐grafted SAMs whose interfacial dipole drastically responds to the specific interaction with target molecules, the diode‐like device could be developed as a new sensing platform.^[^ ^36^, ^37^ ^]^

## 3 Conclusion

In summary, we demonstrated that incorporating an interfacial dipole through SAM deposition can significantly impact the electronic properties of ultrathin semiconductors, resulting in the p‐ to n‐type inversion of the 50 nm silicon. The type and carrier density of the 50 nm silicon were determined by the direction and strength of the interfacial dipole of deposited SAM layers, which were verified through the Hall measurements and surface analytic techniques such as UPS, XPS, and KPFM. These results show that in the case of the ultrathin semiconductor, SAM deposition as an interface engineering approach has the potential for a simple alternative to the traditional impurity doping technique, which has difficulty in regulating the unstable performance due to the randomly distributed dopant in nanoscale semiconductors.

Furthermore, we engineered the lateral electronic junction of the 50 nm silicon by regioselective SAM deposition of PFOTS and APTE molecules, which have an opposite interfacial dipole and thereby induce p‐ and n‐type in each silicon region. The 50 nm silicon with the lateral electronic junction exhibited rectification behavior and operated like a p‐n diode, which is fabricated not by the chemical doping into the atomic structure of the semiconductor but by the simple surface treatments. We also confirmed that the rectification ratio was sensitively changed according to the pH of the treating solutions. These results imply that the PFOTS/APTES‐deposited 50 nm silicon could be directly used as a pH sensor. By introducing the various probe‐grafted SAMs, whose interfacial dipole significantly responds to the specific interaction with targets, the ultrathin silicon treated with the regioselective SAM deposition could be developed into a new sensing platform with high sensitivity and low limit of detection.

## 4 Experimental Section

### Preparation for the Silicon Substrate

A silicon‐on‐insulator (SOI) wafer (Biotain Crystal) was used as a silicon substrate, which is composed of a 50 nm silicon device layer, a 375 nm SiO_2_ buried oxide, and a 525 µm silicon handle wafer. Except for the thickness, the specifications of the two silicon layers are identical, with p‐type (B‐doped), resistivity (1–20 ohm·cm), and orientation (<100>). The 4‐inch SOI wafer was cut into 1 × 1.2 cm^2^ pieces and ultrasonicated sequentially with detergent, distilled water (DIW), acetone, and isopropyl alcohol (IPA) for 15 min each. Gold electrodes, each with a size of 1 × 4 mm^2^, were thermally evaporated as source and drain, and the distance between them was 7 mm.

### Self‐Assembled Monolayer (SAM) Deposition on the Silicon Surface

All of the silicon substrate was treated with ultraviolet ozone treatment for 10 min before the SAM deposition. i) (3‐Aminopropyl)triethoxysilane (APTES) deposition was conducted through liquid phase dipping. The UVO‐treated silicon was dipped in 1% v/v APTES in toluene at 85 °C for 0.5 and 1 h. The APTES‐treated silicon was rinsed with toluene and ethanol sequentially and dried under N_2_ flow. ii) Triethoxy(1H,1H,2H,2H‐perfluoro‐1‐octyl)silane (PFOTS) deposition was conducted through chemical vapor deposition. The UVO‐treated silicon was placed in a Teflon chamber with a slide glass containing 30 µL PFOTS at 200 °C for 0.5, 1, and, 1.5 h followed by rinsed with IPA and dried under N_2_ flow.

### Regioselective PFOTS and APTES Deposition

The PFOTS deposition for 1.5 h was conducted on the 50 nm silicon in the same manner as described above. The UVO treatment for 30 min was conducted on the PFOTS‐treated silicon, half of which was covered with a metal mask to prevent light irradiation. As shown in Figure S4, Supporting Information, the contact angle on the UV‐exposed surface shows *ca* 0° indicating that all the PFOTS molecules were removed and the silanol surface was formed. Additionally, the wetting of water droplets at the center of the surface demonstrates a clear boundary formation between the PFOTS‐treated surface and the silanol surface. Subsequently, APTES was deposited for 1 hr using the same method mentioned above.

### Characterization of the Silicon

Hall measurement was conducted to investigate the semiconductor type and majority carrier density by Hall effect measurement system (HL5500, Nanometrics). UPS (NEXSA, Thermofisher) was conducted to characterize the work function shift by the APTES and PFOTS deposition on the silicon. KPFM (NX‐100, Park System) was conducted to characterize the work function variation on the regioselective APTES and PFOTS deposited silicon surface. The surface chemical states of the APTES‐deposited silicon with the ion pairing were characterized by XPS (NEXSA, Thermofisher). Electrical characterization on all silicon devices was conducted using Keysight 2902B source measure units.

## Conflict of Interest

The authors declare no conflict of interest.

## Supporting information



Supporting Information

## Data Availability

The data that support the findings of this study are available from the corresponding author upon reasonable request.
