# Supplementary material for: Lateral Electronic Junction of a Single Ultrathin Silicon Induced by Interfacial Dipole of Self‐Assembled Monolayer
Source: Adv Sci (Weinh). 2024 Sep 9;11(41):2403970. doi: 10.1002/advs.202403970 (PMC11538666; doi:10.1002/advs.202403970)
Supplement: Supplementary file 1 — Supporting Information [file ADVS-11-2403970-s001.docx]

Supporting Information

**Lateral electronic junction of a single ultrathin silicon induced by interfacial dipole of self-assembled monolayer**

*Junghyup Han^‡^, Won Hyung Lee^‡^, Junwoo Park, Huding Jin, Yong Hyun Cho, Seungyeon Yu, Lianghui Li, Jaewon Lee, Gunhoo Woo, Taesung Kim, Youn Sang Kim**

**Table-of-contents:**

**Figure S1.** ARXPS analysis of the APTES-deposited 50 nm silicon.

**Figure S2.** Contact angle measurement of the UVO-treated silicon surface and the subsequently annealed silicon surface.

**Figure S3.** Contact angle measurement of the PFOTS-deposited 50 nm silicon surface according to the PFOTS deposition time.

**Figure S4.** Regioselective PFOTS and APTES deposition on the 50 nm silicon.

**Figure S5.** XPS analysis of PFOTS-deposited silicon surface treated with NaCl solutions of pH levels (3, 7, 11).

**Supporting Note 1.** Depth profile of the APTES-deposited 50 nm silicon through ARXPS analysis.


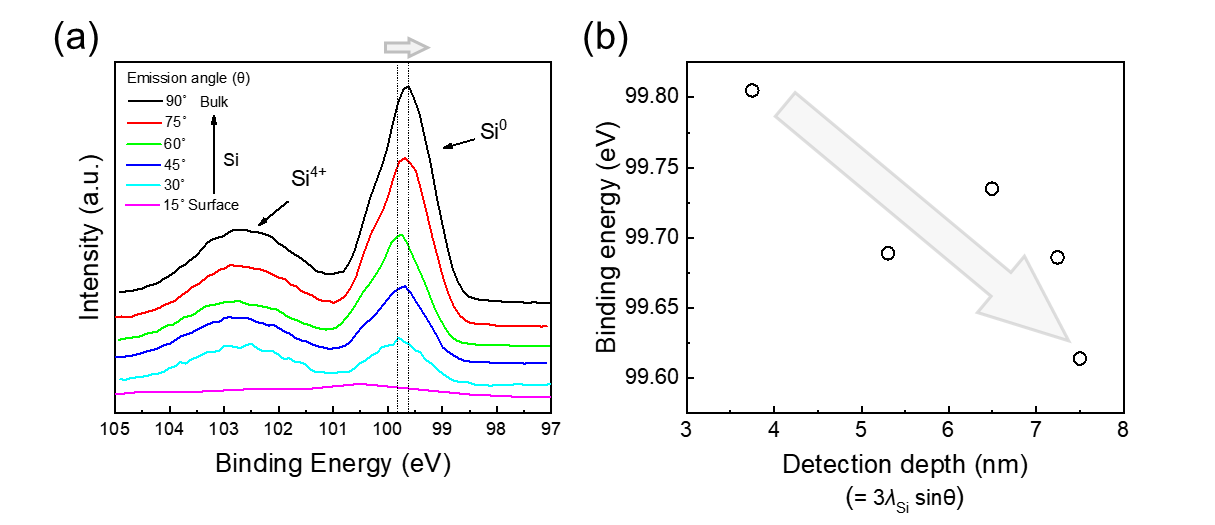
**Figure S1.** ARXPS analysis of the APTES-deposited 50 nm silicon. (a) Si 2p XPS spectra according to the emission angle (*θ*). Each dashed line is the binding energy of Si^0^ 2p at emission angle of 30 ˚ and 90˚, respectively. (b) Depth profile of the binding energy of Si^0^ 2p peaks indirectly indicating the decrease of electron density as deeper.


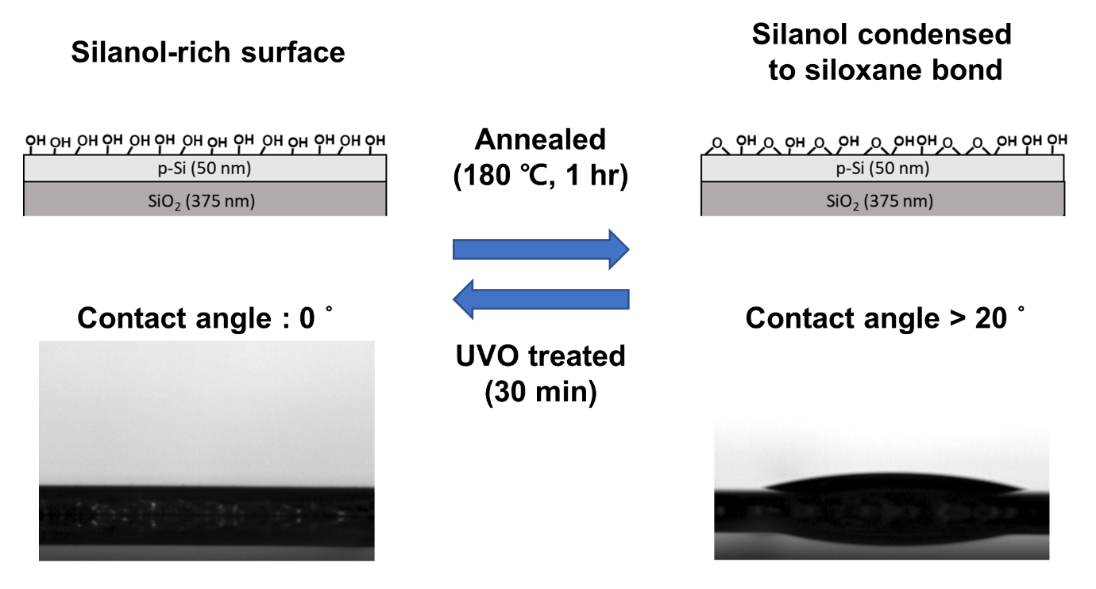


**Figure S2.** Contact angle measurement of the UVO-treated silicon surface and the subsequently annealed silicon surface. The schematic image depicts the silanol-to-siloxane interconversion on the silicon surface induced by the iterative alternation of UVO treatment and annealing process. The contact angle of the UVO-treated silicon surface measured as *ca*. 0 ˚, attributed to the abundance of hydrophilic silanol groups. After the annealing process, the contact angle of the silicon surface exceeds 20 ˚, which indicates that a few of the silanol groups were converted to siloxane bonds, rendering the surface less hydrophilic.


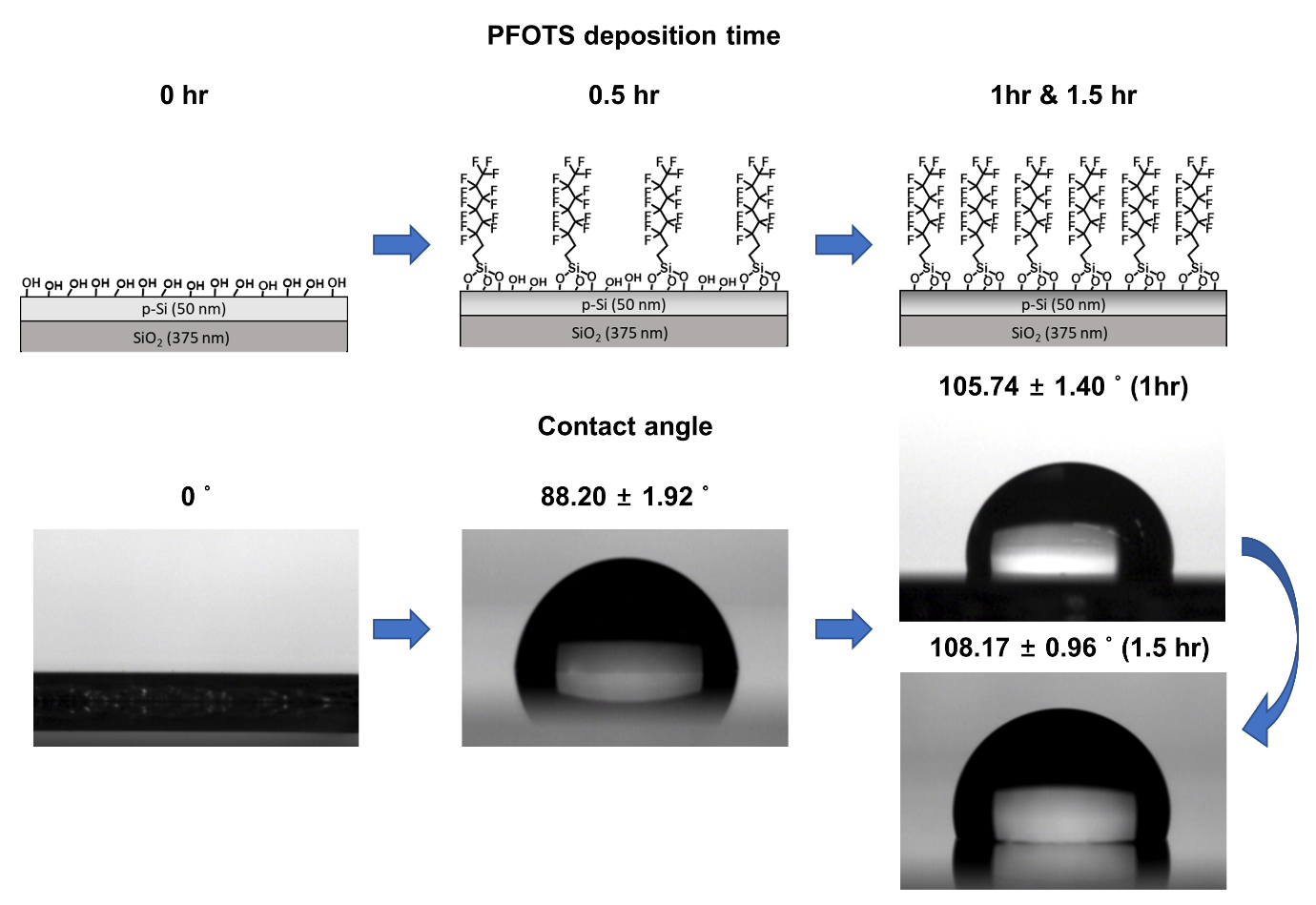


**Figure S3.** Contact angle measurement of the PFOTS-deposited 50 nm silicon surface according to the PFOTS deposition time. The schematic image illustrates that the silicon surface becomes increasingly packed with PFOTS molecules as the PFOTS deposition time increases. The contact angle increases and then saturates at *ca*. 110 ˚, indicating that the silicon surface is fully packed with PFOTS molecules.


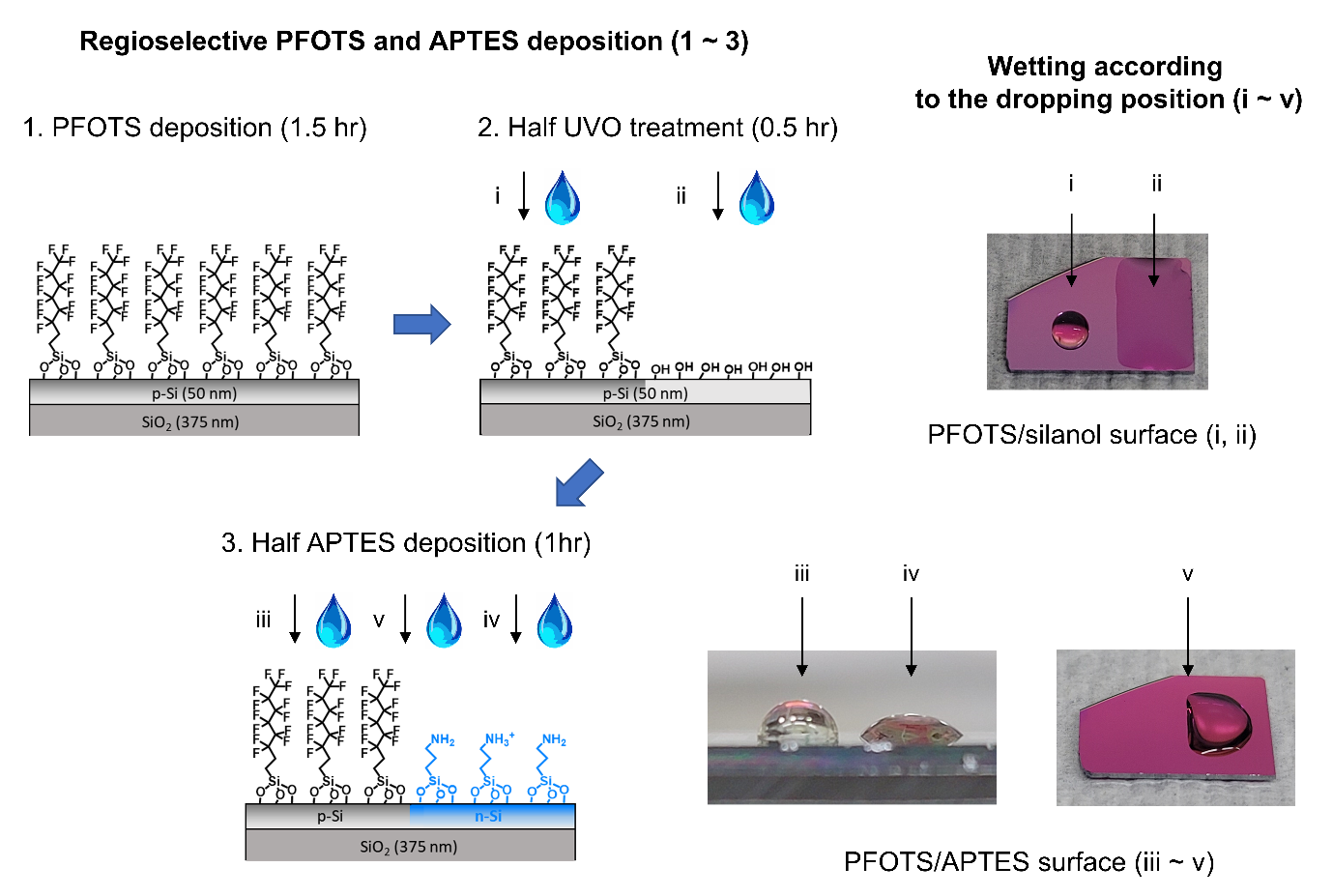


**Figure S4.** Regioselective PFOTS and APTES deposition on the 50 nm silicon. Wetting properties of the silicon surface at each fabrication step show the successful formation and preservation of each SAM-deposited surface. i, The PFOTS-deposited surface was preserved by blocking the UV light with a metal mask during the UVO treatment. ii, Deposited PFOTS molecules were eliminated by the UVO treatment. iii, Contact angle of the PFOTS-deposited surface is not changed after the Half UVO treatment indicating that APTES is not additionally deposited on that PFOTS-deposited surface. iv, Increase in the contact angle of the APTES-deposited surface shows the successful APTES deposition. v, Wetting occurred exclusively on the hydrophilic APTES-deposited surface along the boundary, demonstrating the well-formed boundary between PFOTS- and APTES-deposited surfaces


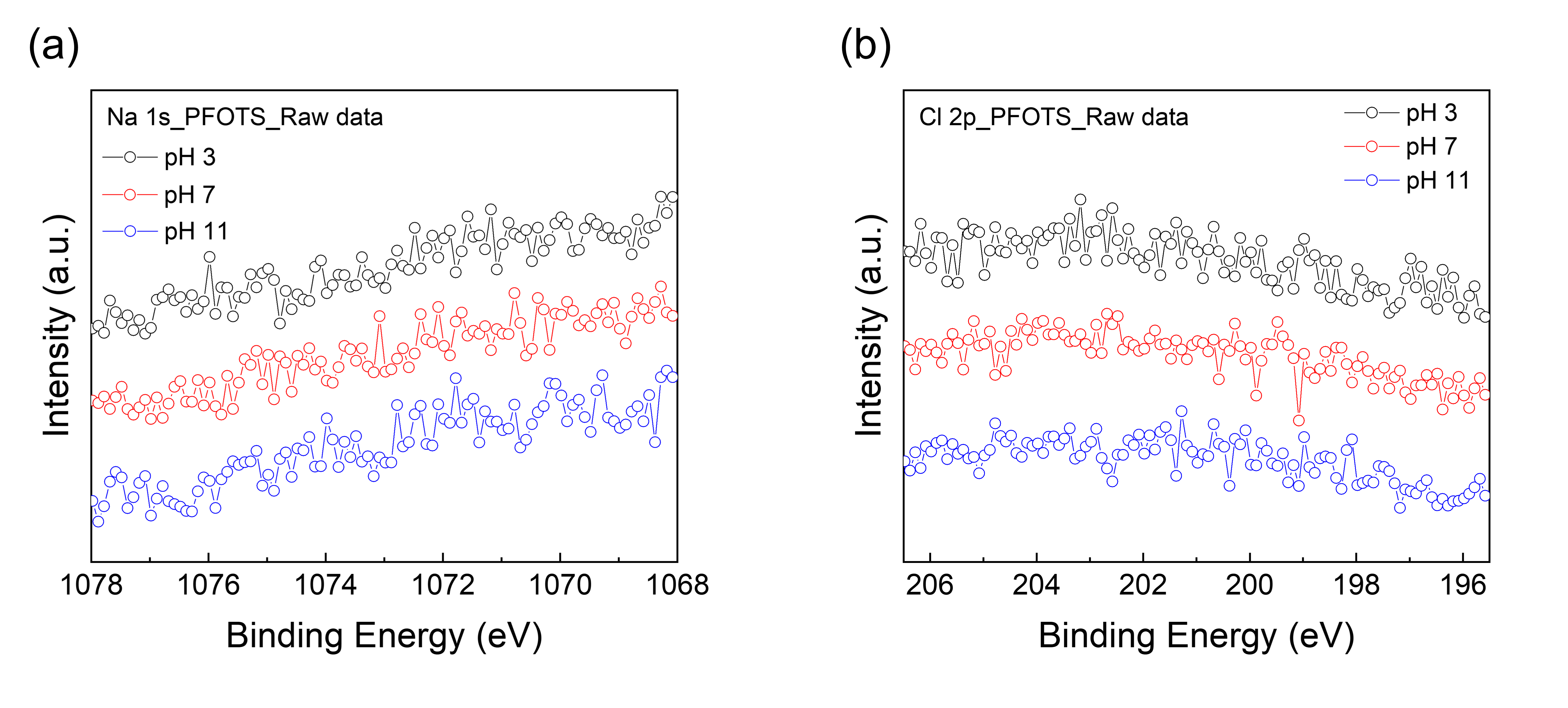


**Figure S5.** XPS analysis of PFOTS-deposited silicon surface treated with NaCl solutions of pH levels (3, 7, 11). No peak was observed in (a) Na 1s and (b) Cl 2p XPS spectra indicating that the Na^+^ and Cl^-^ ions are not paired with the PFOTS molecules in all pH conditions. Therefore, the interfacial dipole of the PFOTS-deposited layer is not affected by the ion pairing method using NaCl solutions.

**Supporting Note 1.** Depth profile of the APTES-deposited 50 nm silicon through ARXPS analysis.

We can indirectly confirm the effect of the APTES on the carrier density of silicon by measuring the binding energy of Si^0^ (elemental silicon) 2p ^[S1,S2]^. The conventional method for XPS depth profiling using ion sputtering is not applicable, thus we chose a non-destructive approach, Angle-Resolved XPS (ARXPS), which allows us to observe the binding energy at different depths by adjusting the emission angle of the electrons. We analyzed the APTES-deposited 50 nm silicon through ARXPS and observed the binding energy shift of Si^0^ 2p peak at different emission angles, related to the electron density at each depth ^[S3,S4]^. As shown in Figure S1a, the Si 2p spectrum features peaks corresponding to the oxide (Si^4+^, 102.7eV) originated from the native SiO_2_ and anchoring groups of APTES, and the elemental silicon (Si^0^, 99.6–99.8 eV), of which binding energy is higher in case of the n-type silicon than p-type silicon^[S5,S6]^. Detection depth depends on the emission angle (*θ*) in a simple relation of $d=3\lambda\sin\theta$, where λ is the inelastic mean free path of photoelectrons. The value of λ is 2.5 nm for photoelectrons of Si 2p, as calculated by TPP-2M method21 in NIST’s database^[S7]^. The binding energy of Si^0^ 2p peak decreases with the increase of detection depth indirectly indicating that the electron density decreases with increasing depth (*θ* = 90˚) (Figure S1b). Thus, we confirmed that the effect of the interfacial dipole layer of APTES, which induces p- to n-type inversion influences up to a depth of at least 7.5 nm though the effect decreases with increasing depth from the surface.

**Reference of Supporting Note 1**

[S1] F. J. Himpsel, G. Hollinger, R. A. Pollak, *Phys. Rev. B* **1983**, *28*, 7014.

[S2] D. C. Gleason-Rohrer, B. S. Brunschwig, N. S. Lewis, *J. Phys. Chem. C* **2013**, *117*, 18031.

[S3] C. R. Brundle, G. Conti, P. Mack, *J. Electron Spectros. Relat. Phenomena* **2010**, *178*–*179*, 433.

[S4] Y. Zhao, H. Gao, R. Huang, Z. Huang, F. Li, J. Feng, Q. Sun, A. Dingsun, H. Yang, *Sci. Rep.* **2019**, *9*, 16969.

[S5] W. M. Lau, *J. Appl. Phys.* **1989**, *65*, 2047.

[S6] H. Sezen, S. Suzer, *J. Chem. Phys.* **2011**, *135*, DOI 10.1063/1.3652964.

[S7] C. K. Powell, A. Jablonski, “NIST IMFP database,” can be found under https://www.nist.gov/system/files/documents/srd/SRD71UsersGuideV1-2.pdf, **2010**.
